# Supplementary material for: Diversity? Great for Most Just Less So for Me: How Cognitive Abstraction Affects Diversity Attitudes and Choices
Source: Behav Sci (Basel). 2025 Apr 27;15(5):585. doi: 10.3390/bs15050585 (PMC12109399; doi:10.3390/bs15050585)
Supplement: Supplementary file 1 [file behavsci-15-00585-s001.zip › behavsci-3398716-supplementary.pdf]

Supplemental Materials

Study 1

Instructions: Thank you for taking part to this study. We are interested in people's perception about groups.

Diversity Attitudes

1 (Not at all) to 7 (Very much)

1. I believe that *most groups/ groups to which I belong* benefit from the involvement of people with different backgrounds.
2. I think that for *most groups/ groups to which I belong* having members with different backgrounds is helpful.
3. I think that *most groups/ groups to which I belong* should be characterized by members' diversity.
4. I believe it can be very problematic if *most groups/ groups to which I belong* include different people.
5. I think that *most groups/ groups to which I belong* should contain similar people.
6. I think that *most groups/ groups to which I belong* could be more attractive if people in them are different.

Perspective Taking

1 (Not at all) to 7 (Very much)

1. Before criticizing somebody, I try to imagine how I would feel if I were in their place.
2. If I'm sure I'm right about something, I don't waste much time listening to other people's arguments.
3. I sometimes try to understand my friends better by imagining how things look from their perspective.
4. I believe that there are two sides to every question and try to look at them both.
5. I sometimes find it difficult to see things from the "other guy's" point of view.
6. I try to look at everybody's side of a disagreement before I make a decision.
7. When I'm upset at someone, I usually try to "put myself in his shoes" for a while.

Study 2

Diversity Attitudes

1 (Strongly disagree) to 7 (Strongly agree)

1. *Most companies/ The company I currently work for* should hire people from different backgrounds.
2. *Teams within most companies/ My teams at the company I currently work for* should always contain both males and females.
3. *In most companies, teams/ My teams at the company I currently work for* can benefit from the involvement from people with different ethnic backgrounds.
4. *Within most companies, teams/ At the company I currently work for, my teams* with members from different backgrounds are often a recipe for trouble.
5. *In most companies/ At the company I currently work for*, a mix of people from different backgrounds helps doing a task well.
6. *In most companies, creating teams with/ At the company I currently work for, being involved in mixed-teams where* members have different views and opinions is beneficial for productivity.

Participant Profile

1. Please indicate the gender type that you most identify with.
   - Male
   - Female

2. Please indicate your age.
   - 20 - 35
   - 36 - 45
   - 46 - 55
   - 56 - 65
   - > 65

3. Please indicate your highest obtained academic degree.
   - High School
   - Bachelor's
   - Master's
   - Ph.D
   - None

4. Please indicate which ethnic group you most identify with.
   - African American
   - Arabic
   - Asian
   - Caucasian
   - Hispanic/Latino

5. Please indicate which culture you most identify with. (For example, the one in which you grew up)
   - American (US)
   - Arabic
   - Asian
   - European (East)
   - European (North/West)
   - European (South/Mediterranean)
   - South American (Latin)

6. Please indicate which political group you most identify with.
   - Left wing
   - Right wing

The following two questions concern two personality dimensions of the 'Big Five' personality inventory: extroversion and agreeableness.

Please indicate which the following is most applicable to you.

7. In general, I am...
   - Cooperative and friendly: a real team-player who likes to work together and prefers an harmonious environment
   - Individualistic and analytical: I have mind of my own and I am not afraid to act accordingly, even when others disagree

8. In general I am...
   - Outgoing, open and not afraid to speak my mind among other people
   - Soft-spoken and reserved. I prefer to keep a low profile and stay in the background

Candidate Profiles

Candidate 1.
Male, 25 years old with a master's degree and 1 year of experience. He is from African American descent and grew up in the United States. He is a left wing voter, and would describe himself as an introverted person who enjoys working together in a team.

Candidate 2.
Female, 40 years old with a Ph.D and 14 years of experience. She is from Arabic descent and grew up in the Egypt. She is a lef wing voter, and would describe herself as an introverted person who is not afraid to stick to her own opinion, even when others disagree with her.

Candidate 3.
Male, 50 years old with a Bachelor's degree and 10 years of experience. He is from

Asian descent and also grew up in Japan. He is a right wing voter, and would describe himself as an outspoken and energetic person, who will speak his mind and follow his own path.

Candidate 4.
Male, 65 years old with a high school degree and 40 years of experience. He is caucasian and grew up in Scandinavia. He is a right wing voter, and would describe himself as a softspoken person who prefers to keep the peace over standing by his own opinion.

Candidate 5.
Female, 35 years old with a Bachelor's degree and 6 years of experience. She is caucasian and grew up in Hungary. She is a right wing voter, and would describe herself as an extraverted people's person, who does not like to put her foot down and is inclined to give in to other people.

Candidate 6.
Female, 56 years old with a Master's degree. She is from hispanic descent and grew up in Spain. She is a left wing voter and would describe herself as a reserved person, who is not easily persuaded by other people's opinions.

Candidate 7.
Male, 45 years old with a high school degree. He is from latin descent and grew up in Mexico. He is a right wing voter and would describe himself as an outgoing person, who does not mind to make a compromise if this is asked of him.

Candidate 8.
Female, 67 years old with a Master's degree. She is of African America descent and grew up in The Netherlands. She is a left wing voter and would describe herself as an outspoken person who is not afraid to stand her ground when other people try to convince her of their opinion.

Study 3

How manipulation

For everything we do, there always is a process of how we do it. Moreover, we often can follow our broad life-goals down to our very specific behaviors. For example, like most people, you probably hope to find happiness in life. How can you do this? Perhaps living comfortably, or enjoying yourself, can help. How can you do these things? Perhaps by buying things you want or need. How do you buy things you want or need? Maybe by earning extra money. How do you earn extra money? In some cases, such as today, you complete a survey.

This thought exercise is intended to focus your attention on how you do the things you do. For this thought exercise, please consider the following activity: 'improving and maintaining your physical health'

In the spaces below, please list three ways in which you can improve and maintain your physical health.

1. One way in which I can improve and maintain my physical health is by...
2. Another way in which I can improve and maintain my physical health is by...
3. An additional way in which I can improve and maintain my physical health is by...

Why manipulation

For everything we do, there always is a reason why we do it. Moreover, we often can trace the causes of our behavior back to broad life-goals that we have. For example, you are currently completing a survey. Why are you doing this? Perhaps to earn extra money. Why are you earning extra money? Perhaps to buy things that you want or need. Why buy things that you want or need? Maybe because you want to live comfortably, or because you want to enjoy yourself. And perhaps you wish to live comfortably or enjoy yourself because you feel that doing so can bring you happiness in life.

This thought exercise is intended to focus your attention on why you do the things you do. For this thought exercise, please consider the following activity: 'improving and maintaining your physical health'

In the spaces below, please list important life goals which improving and maintaining your physical health can help you meet.

1. One important life goal which improving and maintaining my physical health can help me meet is...
2. Another important life goal which improving and maintaining my physical health can help me meet is...
3. An additional important life goal which improving and maintaining my physical health can help me meet is...

Diversity Attitudes (see Study 1)

Perspective Taking (see Study 1)

System Justification

1 (Strongly disagree) to 7 (Strongly agree)

1. In general, I find American society to be fair.
2. In general, the American political system operates as it should.
3. American society needs to be radically restructured.
4. The United States is the best country in the world to live in.
5. Most US policies serve the greater good.
6. Everyone has a fair shot at wealth and happiness in America.
7. American society is getting worse every year.
8. American society is set up so that people usually get what they deserve.

Social Dominance Orientation

1 (Strongly disagree) to 7 (Strongly agree)

1. Some groups of people are just more worthy than others.
2. It's OK if some groups have more of a chance in life than others.
3. If certain groups of people stayed in their place, we would have fewer problems.
4. Inferior groups should stay in their place.
5. It would be good if all groups could be equal.
6. We should increase social equality.
7. We would have fewer problems if we treated different groups more equally.
8. No one group should dominate in society.

Attention Check

Recent research on perceptions of groups shows that choices are affected by context. Differences in how people feel, their previous knowledge and experience, and their environment can affect choices. We are interested in whether you actually take the time to read directions. Please ignore the job attributes below and instead select four as your answer for all attributes.

Please rate the extent to which each job attribute is important to you.

1 (Not at all) to 7 (Very much)

1. Educational opportunities
2. Diverse colleagues
3. Challenging tasks

Study 4

Diversity Attitudes (see Study 1)

Pros versus Cons Scale

1 (Not at all) to 7 (Very much)

1. The upsides of diversity tend to outweigh the downsides of diversity.
2. Managing the downsides of diversity is worth it in order to capture the benefits of diversity.
3. There is too much work required to capture the benefits of working with diverse people.

Pros List

List as many pros in the spaces below that come to mind when you think about diversity in *most groups and teams/ groups and teams to which you belong.*

Cons List

List as many cons in the spaces below that come to mind when you think about diversity in *most groups and teams* / *groups and teams to which you belong*.

Perspective Taking (see Study 1)

System Justification (see Study 3)

Social Dominance Orientation (see Study 3)

Attention Check (see Study 3)

Study 5

Diversity Attitudes (see Study 2)

Participant Profile (see Study 2)

Candidate Profiles

Candidate 1.
Male, 25 years old with a master's degree and 1 year of experience. He is from African American descent and grew up in the United States. He is a left wing voter, and would describe himself as an introverted person who enjoys working together in a team.

Candidate 2.
Female, 40 years old with a Ph.D and 14 years of experience. She is from Arabic descent and grew up in Egypt. She is a left wing voter, and would describe herself as an introverted person who is not afraid to stick to her own opinion, even when others disagree with her.

Candidate 3.
Male, 50 years old with a Bachelor's degree and 10 years of experience. He is from Asian descent and also grew up in Japan. He is a right wing voter, and would describe himself as an outspoken and energetic person, who will speak his mind and follow his own path.

Candidate 4.
Male, 65 years old with a high school degree and 40 years of experience. He is Caucasian and grew up in the United States. He is a right wing voter, and would describe himself as a soft-spoken person who prefers to keep the peace over standing by his own opinion.

Candidate 5.
Female, 35 years old with a Bachelor's degree and 6 years of experience. She is Caucasian and grew up in Hungary. She is a right wing voter, and would describe herself as an extraverted people's person, who does not like to put her foot down and is inclined to give in to other people.

Candidate 6.
Female, 56 years old with a Master's degree and 10 years of experience. She is from Hispanic descent and grew up in Spain. She is a left wing voter and would describe herself as a reserved person, who is not easily persuaded by other people's opinions.

Candidate 7.
Male, 45 years old with a high school degree and 15 years of experience. He is from Latin descent and grew up in Mexico. He is a right wing voter and would describe himself as an outgoing person, who does not mind making a compromise if this is asked of him.

Candidate 8.
Female, 67 years old with a Master's degree and 20 years of experience. She is of African American descent and grew up in England. She is a left wing voter and would describe herself as

an outspoken person who is not afraid to stand her ground when other people try to convince her of their opinion.

Pros List

List as many pros in the spaces below that come to mind when you think about diversity in *teams at most companies/ your own teams at the company you currently work for*.

Cons List

List as many cons in the spaces below that come to mind when you think about diversity in *teams at most companies/ your own teams at the company you currently work for.*

Social Dominance Orientation (see Study 3)

Attention Check (see Study 3)
